# Handheld Spatially Offset Raman Spectroscopy for rapid non-invasive detection of ethylene glycol and diethylene glycol in medicinal syrups

**DOI:** 10.1016/j.jpba.2025.117031

**Published:** 2025-06-14

**Authors:** Sneha Banerjee, Sara Mosca, Isabelle Legge, Bevin Gangadharan, John Walsby-Tickle, Benediktus Yohan Arman, Robert Stokes, Tehmina Bharucha, Michael Deats, Hamid A. Merchant, James McCullagh, Nicole Zitzmann, Céline Caillet, Paul N. Newton, Pavel Matousek

**Affiliations:** a Central Laser Facility, Research Complex at Harwell, STFC Rutherford Appleton Laboratory, UKRI, Harwell Campus, OX11 0QX, UK; b Department of Chemistry, University of Oxford, Oxford OX1 3TA, UK; c Department of Biochemistry, University of Oxford, Oxford OX1 3QU, UK; d Kavli Institute for Nanoscience Discovery, University of Oxford, Oxford OX1 3QU, UK; e Agilent Technologies LDA UK, Becquerel Avenue, Didcot OX11 0RA, UK; f Medicine Quality Research Group, NDM Centre for Global Health Research, Nuffield Department of Medicine, University of Oxford, Oxford OX3 7LG, UK; g Mahidol-Oxford Tropical Medicine Research Unit, Faculty of Tropical Medicine, Mahidol University, Bangkok 10400, Thailand; h Infectious Diseases Data Observatory, Centre of Tropical Medicine & Global Health, Nuffield Department of Medicine, University of Oxford, Oxford OX3 7LG, UK; i Department of Bioscience, School of Health, Sport and Bioscience, University of East London, Water Lane, London E15 4LZ, UK

**Keywords:** Spatially Offset Raman Spectroscopy, Medicinal syrups, Substandard, Falsified medicines, Supply chain, Diethylene glycol, Ethylene glycol, Propylene glycol, Glycerol

## Abstract

We investigate the potential of Spatially Offset Raman Spectroscopy (SORS) as a rapid, non-invasive screening tool deployable in the field to detect diethylene glycol (DEG) and ethylene glycol (EG) in medicinal syrups within closed containers. Measurements were performed on neat propylene glycol (PG) and glycerol, key components of many medicinal syrups, as well as marketed medicinal syrup formulations spiked with DEG and EG at various concentration levels to assess the technique’s limit of detection in real-life samples. SORS was able to detect these down to ~0.5 % concentration level in neat PG for both DEG and EG compounds and ~1 % concentration level for DEG and EG in neat glycerol. The DEG and EG detection thresholds for the marketed formulations measured through original bottles was ~1 %, for Benylin (active ingredient: Glycerol) and Piriteze (active ingredient: Cetirizine Hydrochloride). For Calpol (active ingredient: Paracetamol) the detection limit was higher, ~2 % for EG and ~5 % for DEG. Although not reaching the International Pharmacopeial 0.1 % detection threshold currently required for purity checks for human consumption, the method can still be used to detect products where DEG or EG has been wrongly used instead of PG or glycerol or if present in large quantities. The technique could also be used for raw material identification testing to ensure no mislabelling has occurred in pre-production stages and as a screening device in distribution chains to detect major deviations from permitted content in non-diffusely scattering, clear formulations, to help prevent serious adverse outcomes, such as acute renal failure and deaths.

## 1 Introduction

Preventing, detecting, and responding to substandard and falsified medicines is of critical importance for ensuring the safety, effectiveness, and public trust in medical products [1]. Some of the gravest incidents in recent years have been multiple outbreaks of poisoning due to diethylene glycol (DEG) and ethylene glycol (EG) contained in paediatric syrups, resulting in many hundreds of deaths worldwide, mostly children [2–4]. WHO has raised alerts of contaminations in cough [5,6], paracetamol [7] and cetirizine [8] syrups. The most commonly recommended reference assay is gas chromatography with flame ionisation detection (GC-FID) (International Pharmacopeia, 2023). Such reference analytical tests are expensive, complicated to use and typically only available in few specialist laboratories with delayed access, time and cost implications. Fourier transform mid-infrared and near-infrared spectroscopy have been evaluated for this use although they required decanting of the sample [9]. The most practical current screening method is thin layer chromatography (TLC), recommended by WHO, but it has not been reported to reach down to the 0.1 % upper limit of acceptability (International Pharmacopeia 2023). This approach is low cost, but it requires some basic laboratory infrastructure and analytical skills. The invasive nature of this analysis (requiring bottles to be opened) hampers its wider deployment in the field.

The provision of more autonomous, rapid and portable diagnostic techniques deployable at different locations within medical supply chains and ideally deployable without need to open the containers is therefore of very high importance. Here we assess the capability of Spatially Offset Raman Spectroscopy (SORS) to provide a rapid screening of such products through unopened containers [10–12]. The measurements were performed on neat propylene glycol (PG) and neat glycerol, key components of many medicinal syrups, as well as on marketed final formulations of cough (Benylin Infant), paracetamol (Calpol Infant) and cetirizine (Piriteze) syrups purchased in UK pharmacies and spiked with DEG and EG at various levels. This was to assess the ability of the technique to detect the presence of the concerned toxins in both pre-production, i.e. in incoming raw materials, as well as post-production in finished pharmaceutical products in distribution chains. The SORS technique was recently also applied to differentiation of falsified COVID-19 vaccines through vials on the basis of the chemical identity of excipients and their concentrations [13].

## 2 Experimental

### 2.1 SORS handheld device resolve

The measurements were performed using a commercial handheld SORS instrument (*Resolve*, Agilent Technologies, Oxfordshire, UK) [14]. The spectra were collected with 25 s (zero spatial offset = 1 s x 5 a (acquisitions); spatial offset = 2 s x 10 a) overall acquisition time with an 830 nm excitation laser of 475 mW power. SORS spatial offset was 5.5 mm. The overall measurement time accounting also for automated instrumental calibration and background collection was approximately 1.5 min. The measurements were performed in a dark and air-conditioned temperature stabilised laboratory environment. During the measurements the instrument rested on an optical table. When sampling the marketed finished products, the bottles were partially filled and scanned through a side wall close to their bases to avoid labels. Six measurements at different positions were acquired from each container with the container removed, rotated and repositioned between the measurements.

### 2.2 Samples

Glycerol (Ph. Eur. grade, Sigma-Aldrich, St. Louis, Missouri, United States), Propylene Glycol (Ph. Eur. grade, Sigma-Aldrich), Ethylene Glycol (≥99 %, Sigma-Aldrich), and Diethylene Glycol (for synthesis, Sigma-Aldrich) were obtained from Sigma-Aldrich. The finished products Piriteze, Benylin Infant, and Calpol Infant were purchased in community pharmacies in the UK (Table 1).

While in the International Pharmacopia (IP) protocol the EG-DEG reference solutions are prepared with both EG and DEG in the same single reference solution [15], in our work, we created different reference solutions for EG and DEG. These were prepared by gravimetrically adding EG or DEG to the bulk excipient, PG, glycerol or to liquid marketed finished products, generating 10, 5, 2, 1, 0.5, 0.1 and 0.05 % w/w solutions. Neat solutions of the bulk excipients were also prepared.

**Fig. 1:**
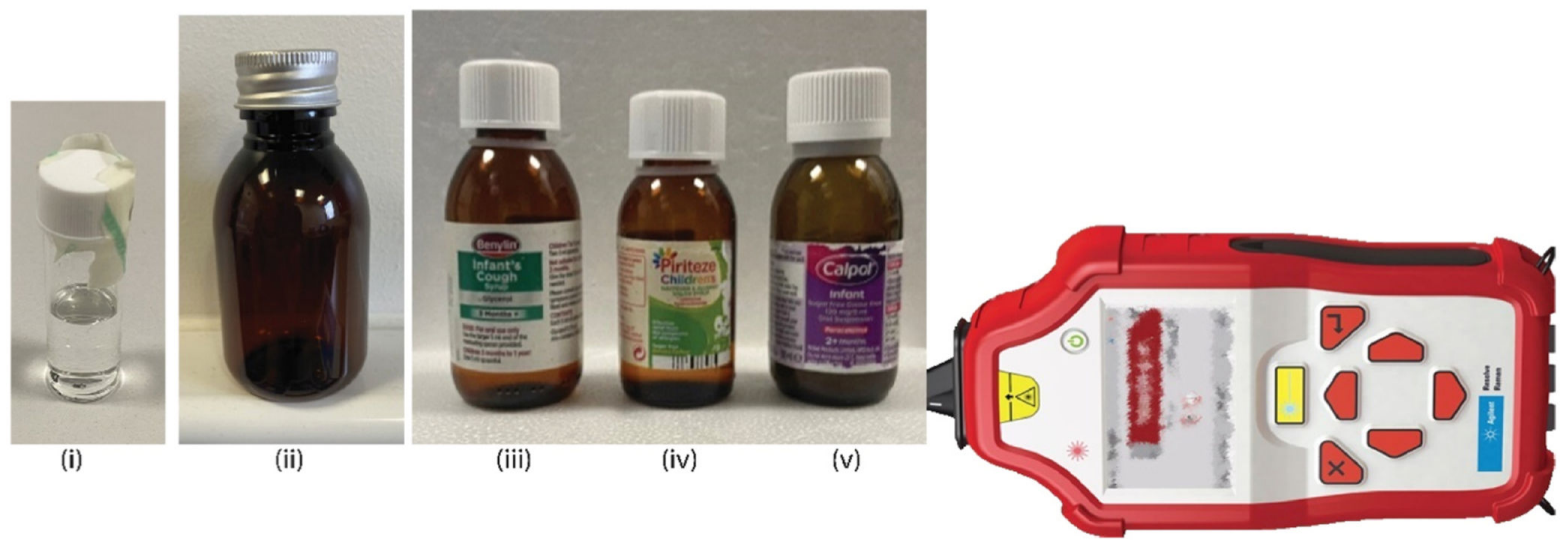
Sample containers used in the study along with a SORS handheld device *Resolve* (not to scale). (i) Clear glass vial (ii) amber plastic (PET) bottle. Original amber glass bottles of (iii) Benylin (iv) Piriteze (v) Calpol.

The samples were measured in standard clear glass vials (Agilent, accessory for Resolve, 15 mm diameter) and amber 100 ml polyethylene terephthalate (PET) plastic medicine bottles with an aluminium cap (50 mm diameter; Ampulla, Cheshire, UK) (Fig. 1). The reference spectra were acquired using SORS through clear glass containers, the PET containers or the original containers.

### 2.3 Data analysis

The Raman spectra were processed manually after exporting them from the Raman device to a computer. They were analysed using a semi-automated SORS container/content signal separation routine involving scaled subtraction of the zero spatial offset spectrum from the spatially offset spectrum. The scaling factor was selected to bring the container fluorescence background at around ~ 675 cm^−1^ approximately to zero in the subtracted spectrum to reveal the Raman spectrum of the vial content alone. The code was written in MATLAB R2019b (MathWorks - Natick, Massachusetts, USA).

Further processing was performed in multivariate analysis package Solo 9.2 (Eigenvector Research Inc.). All the Raman spectra were first truncated below 700 cm^−1^ and above 1600 cm^−1^ to retain chemically the most informative range whilst minimising noisier and less informative parts of the spectra, and a standard multivariate analysis pre-processing was applied. This included background removal (Weighted Least Squares, 2nd order polynomial), spectral smoothing (Savitzky-Golay, width 15 pts and 4th polynomial), standard normal variate normalisation (SNV) and mean-centring.

The six measurements performed on each solution were separated into two sets of spectra (each set containing 3 measurements) used as calibration and prediction data sets.

For the spiked neat solutions (0–5 %) Partial Least Squares (PLS) analysis was used to train and predict individual concentrations, (Solo 9.2.1, Eigenvector Research Inc., Manson, WA, USA) (3 latent variables apart from DEG in glycerol where 5 components were required due to a noisier set of spectra).

For the spiked marketed finished products, the same data treatment and analysis was performed with the exception of using 4 latent variables (both amber PET and original bottles) accounting for more complex formulations being present. For the marketed bottles, 10 % spiking data points were introduced (0–10 %).

## 3 Results and discussion

**Fig. 2:**
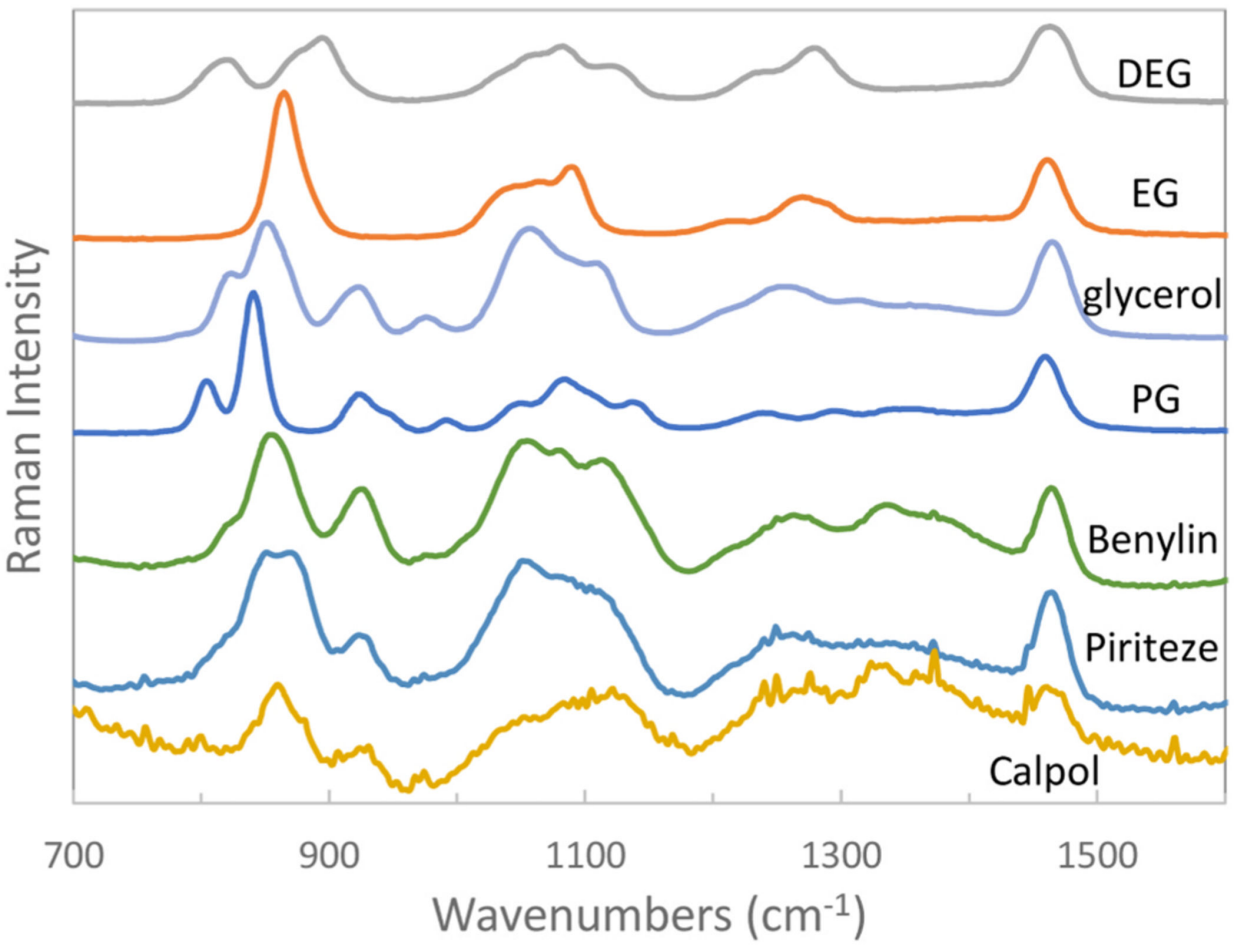
Representative Raman spectra of neat compounds and marketed finished products used in this study (DEG and EG are toxins and PG and glycerol are natural components of many syrups).

First, we acquired the reference Raman spectra of DEG, EG, PG, glycerol and marketed finished products (see Fig. 2). Except for Calpol, the spectra were of high quality. The lower spectral quality of Calpol is attributed to the highly optically diffusely scattering nature of its formulation (all the other formulations were transparent liquids). Calpol is a cloudy (opaque) suspension of paracetamol which gives rise to the lower spectral quality. To our knowledge, the contaminated paracetamol syrups in Indonesia were not cloudy suspensions. Therefore, it is likely that high quality SORS spectra could have been obtained for the Indonesian paracetamol syrups but these could not be tested since suspension-free paracetamol syrups are not available in the UK. Overall, all the spectra exhibited distinct chemical signatures holding good prospects for their differentiation.

Given the high level of concentration of DEG/EG found in syrups in some of the past poisoning incidents, for example with 28.6 % DEG [6] and ~30 % EG[16], it is conceivable that the contamination could have occurred at the production stage of the syrups through using criminally mislabelled DEG/EG as PG/glycerol or other excipients (as DEG/EG is less expensive than PG or glycerol). For EG, contamination up to ~100 % were measured in barrels labelled as PG [17,18]. Furthermore, criminals have previously substituted pharmaceutical grade PG with cheaper industrial grade PG which is less pure and may have DEG/EG above the 0.1 % IP recommended threshold [18]. For this reason, we also investigated the ability of SORS to detect DEG/EG in neat PG and neat glycerol spiked with DEG/EG at various concentration levels. It is also possible that the toxic product could be introduced in supply chains [8] and therefore we also included the measurements of DEG/EG in representative finished products through their original glass amber bottles but also through amber PET plastic bottles since the majority of reported cases have been in similar amber PET plastic bottles.

### 3.1 Detection of DEG/EG in Neat PG and Glycerol

**Fig. 3:**
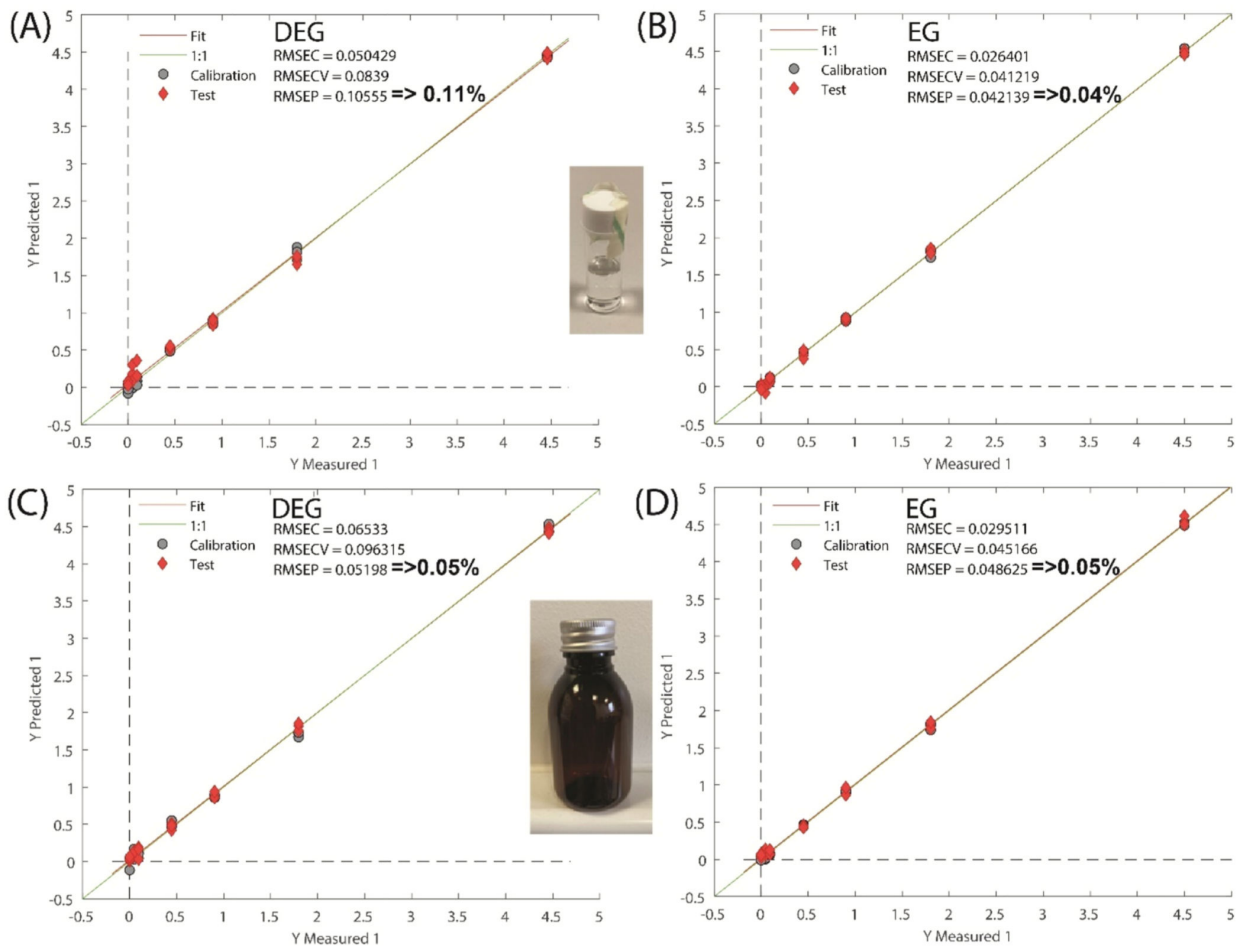
The results of PLS quantification models of DEG (A,C) and EG (B,D) in spiked PG measured through (A-B) glass containers, (C-D) amber PET bottles (RMPSEC – root mean square error of calibration, RMSECV - root mean square error of cross-validation, RMPSEP – root mean square error of prediction).

The spiked neat sample measurements were performed through both glass and amber PET bottles to enable direct comparison between different products and formulations in terms of limits of detection and assess the influence of the container wall. The quantification results are shown in Fig. 3 and Fig. S4. Although the Raman spectra obtained through amber PET containers were of lower quality compared with those through thin-wall clear glass vials (not shown) this did not translate into lower quantification performance, with limits of detection being approximately similar for both types of containers. For example, in all the cases it was possible to detect EG and DEG in PG down to ~ 0.5 % levels. Overall root mean square error of prediction (RMSEP) for EG and DEG was 0.04 % and 0.11 % for glass containers and 0.05 % and 0.05 % for amber PET containers, respectively.

Spiked measurements through amber PET bottles were also performed in glycerol (see Fig. S1 and S2). The limits of detection in neat glycerol was ~1 % for DEG and EG. The root mean square error of prediction (RMSEP) for DEG and EG in glycerol was 0.4 % and 0.3 %, respectively.

### 3.2 Detection of DEG/EG in marketed formulations

The spiked formulations were measured through both the original bottles and amber PET bottles. The latter was done to replicate the bottle used in most reported cases of contaminated syrups and to facilitate direct comparison between different samples (eliminating bottle influence).

**Fig. 4:**
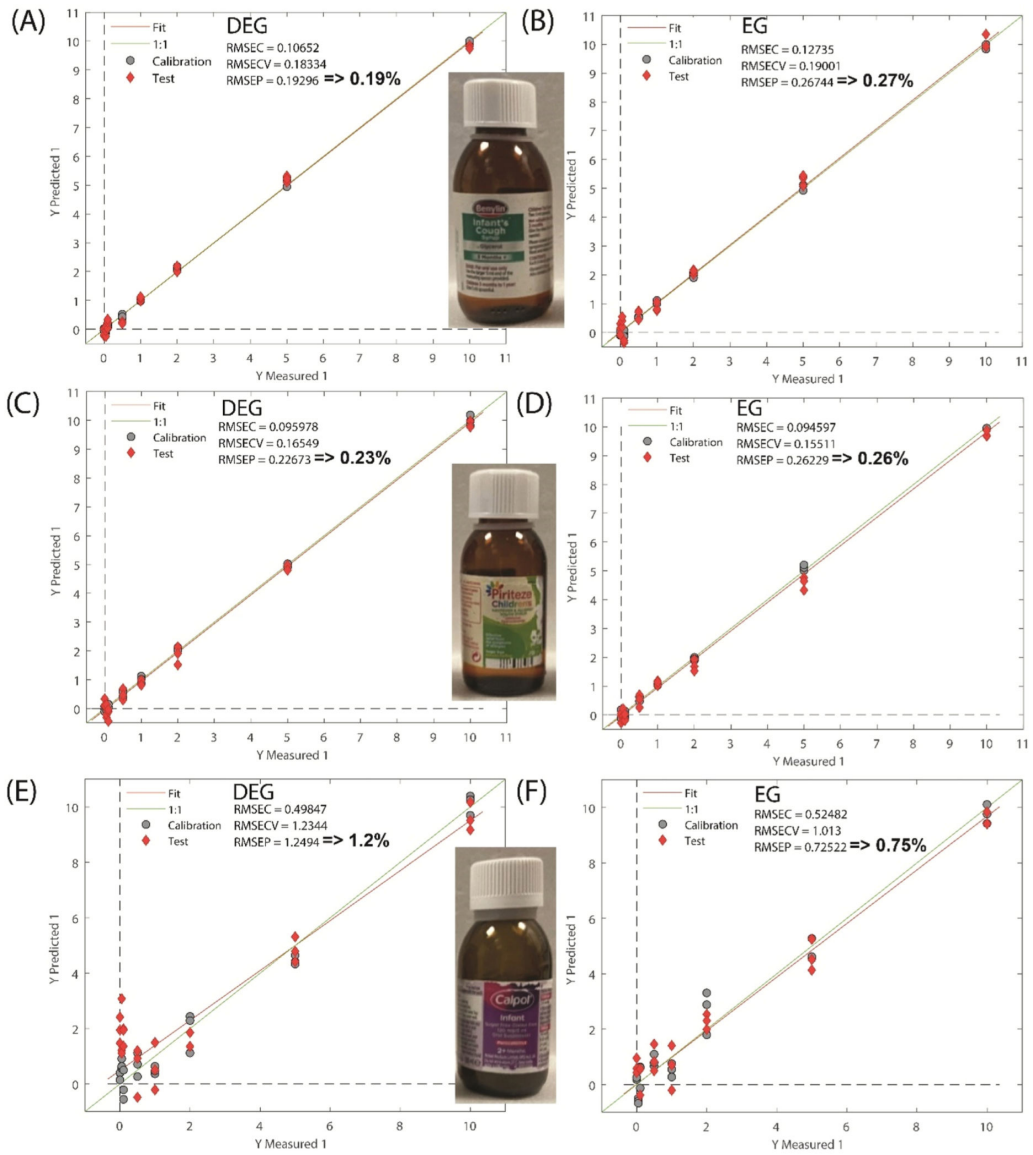
The results of PLS quantification models of DEG (A,C,E) and EG (B,D,F) in spiked marketed full formulations of (A-B) Benylin, (C-D) Piriteze, (E-F) Calpol measured through their original bottles.

Fig. 4 and Fig. S6 illustrate the results of the quantification analysis for DEG and EG in formulations of Benylin, Piriteze and Calpol measured through the original bottles. For Benylin and Piriteze, both DEG and EG were detected down to around 1 %. The RMSEP for Benylin spiked with DEG and EG was 0.19 % and 0.27 %, respectively. The RMSEP for Piriteze spiked with DEG and EG was 0.23 % and 0.26 %, respectively. For Calpol a higher threshold was observed for both DEG and EG, enabling the detection only down to 5 % and 2 %, respectively. The RMSEP for Calpol spiked with DEG and EG was 1.2 % and 0.73 %, respectively. This lower performance we ascribe to the diffusely scattering nature of the liquid formulation, as discussed above. Better detection limits are expected in syrups where paracetamol is not in a cloudy suspension. However, this could not be tested since such paracetamol syrups are not available in the UK. Further optimisation of Calpol sampling parameters could have potentially yielded better results but the specific aim here was to use standard parameters without additional optimisation for all samples in order to assess robustness for field application by a range of non-specialist users.

Somewhat better performance was achieved when sampling the same full formulations through amber PET bottles (see Fig. S3 and S5). For Benylin and Piriteze both DEG and EG were detected down to around 0.5 %. Again, for Calpol a higher threshold was observed enabling the detection only down to 2 % for both DEG and EG. This improved performance with PET plastic bottles is encouraging since in most contamination cases similar amber PET bottles had been used suggesting that if SORS had been used for most of the reported cases the detection limits would have been as low as 0.5 % DEG/EG.

Overall, the SORS performance in the context of this application does not reach the regulatory threshold of 0.1 % DEG/EG [International Pharmacopeia 2023] for purity testing to validate product safety. However, it renders the technique useful as a viable rapid screening device deployable in various points of supply chains and would, for example, have been able to detect the most serious cases of lethal contamination previously reported. More than 200 children died in Indonesia, Uzbekistan and Cameroon and SORS detection could have prevented these deaths since contamination levels [6,16,17] were well above the 0.5–2 % DEG/EG limit of detection for SORS through PET plastic bottles as shown here. The analysis of suspected formulations identified by SORS could be followed with confirmatory tests in a reference laboratory.

Hence the method is particularly well suited for raw material identification testing in pre-production stage on neat samples (not a purity test). This is a regulatory requirement and is performed to ensure no mislabelling happens in pre-production stage in the supply chain of the raw materials entering the production. In fact, many reported incidents are suspected to have taken place at this pre-production stage [18,19]. The risk to patients of SORS not detecting DEG and EG below 1 % and 0.5 %, respectively, in bulk excipient, is likely to be very low as subsequent dilution to manufacturer’s finished formulation is likely to reduce the concentration to below or close to 0.1 %. The testing of marketed formulations is perhaps a somewhat less prospective application given the wide variety of products and the fact that a separate PLS model needs to be built for each brand formulation to ensure optimum prediction performance.

The prediction errors were generally significantly higher than the calibration errors even though the samples in the calibration and validation sets are in effect measurements of the same sample. If the bottles and vials were not moved between the measurements, we would have obtained almost identical spectra. However, as the sample bottles/vials were purposely moved, randomly rotated and repositioned between each measurement, significant errors were observed. This led to small laser and collection beam path alternations (given the relatively large curvature of these containers as well as to presenting a slightly different part of the container wall to the laser beam as the wall thickness and composition are likely to be not entirely uniform). Additionally, exiting laser beams might have also impacted highly heterogeneous bottle labels, where present, at its different positions. Overall, these effects are believed to have led to the errors observed.

Although the measurements were performed in a dark laboratory, for future field deployment a dedicated light-tight compartment tailored to common sample containers could be simply developed. A similar compartment already exists as an instrumental accessory for “*Resolve*” for other types of samples.

Although batch to batch variability has not been investigated in this study, given the tight manufacturing tolerances on concentrations of formulations we would not expect any of these effects to impact prediction accuracies and limits of detections to any significant degree.

Although not a case here, in some cough medicine products colorant could be used. These would not be expected to cause any major issues with the analysis unless absorbing at the laser and Raman detection spectral range (~ 800–1000 nm). If such absorption was present then a test on viability would need to be conducted to establish that the absorption or associated fluorescence emission do not inhibit SORS analysis.

## 4 Conclusions

We have demonstrated that SORS can be used to screen both neat PG, neat glycerol and representative marketed formulations of medicinal syrups for the presence of DEG/EG. Importantly, DEG/EG could be detected through unopened plastic and glass containers down to approximately 0.5 % level in the neat PG and ~1 % concentration level for DEG and EG in neat glycerol through a PET bottle. The detection threshold for the full formulations measured through original bottles was ~1 %, for Benylin and Piriteze spiked with DEG and EG. For Calpol spiked with DEG/EG, the detection limit was higher, ~2 % for EG and ~5 % for DEG, due to the diffusely scattering nature of its formulation, unlike for the other products, hindering its optical scanning. An improved detection limit is, however, expected for other paracetamol liquid products available in different parts of the world, such as those found contaminated in Indonesia, although these were not tested as part of this study as the products were not available in the UK.

The SORS technique reported here could be used as a rapid screening device deployable in various points of existing supply chains to detect the most serious cases of lethal contamination. The detection of suspected formulations from initial screening could be followed by confirmatory tests in a reference laboratory. The method could also be used for raw material identification testing at pre-production stage, to detect any potential mislabelling of incoming raw materials.

It is to be noted that SORS is a potential screening tool and is not a reference assay and in neither case would the technique be suitable for compendial purity testing to ensure product safety at 0.1 % for both DEG and EG. However, reported levels of DEG/EG in contaminated syrups which killed over 200 children in Indonesia, Uzbekistan and Cameroon were considerably higher than the 0.5–2 % limit of detection of SORS through PET plastic bottles confirming that appropriate application of SORS can detect the contaminated products at those levels in preventing serious adverse outcome in children, the fatal acute renal failure.

## Supplementary Material

Supplementary data associated with this article can be found in the online version at 10.1016/j.jpba.2025.117031.

Supplementary File

## Figures and Tables

**Table 1:** Ingredients of the three medicinal syrups analysed by SORS.

Ingredient type	Benylin Infant’s Cough Syrup	Calpol Infant Sugar Free Colour Free Oral Suspension	Piriteze Children’s Hayfever and Allergy Syrup
Active Ingredients	Glycerol, 0.75 ml in every 5 ml	Paracetamol, 120 mg per 5 ml	Cetirizine Hydrochloride, 5 mg per 5 ml
Other Ingredients	Purified water, maltitol liquid (E965), citric acid, hydroxyethylcellulose, sodium citrate and sodium benzoate (E211). 18.24 mg propylene glycol (E1520) in each 5 ml dose.	Maltitol liquid (E965), glycerol, polysorbate 80, sorbitol liquid (E420), methyl parahydroxybenzoate (E218), propyl parahydroxybenzoate (E216), ethyl parahydroxybezoate (E214), microcrystalline cellulose and carmellose sodium, xanthan gum, and purified water. 14.32 mg propylene glycol (E1520) in each 5 ml dose.	Glycerol, propylene glycol, sorbitol (E420), methyl parahydroxybenzoate (E218), propyl parahydroxybenzoate (E216), sodium acetate, acetic acid, saccharin sodium, and purified water.
Flavour	Apple (containing propylene glycol (E1520)	Strawberry (containing propylene glycol (E1520) and benzyl alcohol	Banana
Manufacturer	McNeil Iberica S.L.U., 28805 Madrid, Spain	McNeil Iberica S.L.U., 28805 Madrid, Spain	McNeil Iberica S.L.U., 28805 Madrid, Spain
