# Supplementary material for: Handheld Spatially Offset Raman Spectroscopy for rapid non-invasive detection of ethylene glycol and diethylene glycol in medicinal syrups
Source: J Pharm Biomed Anal. Author manuscript; Available in PMC 2025 Dec 23. (PMC7618519; doi:10.1016/j.jpba.2025.117031)
Supplement: Supplementary File [file EMS211587-supplement-Supplementary_File.docx]

**Supplementary Information of**

**Application of Handheld Spatially Offset Raman Spectroscopy for Rapid Detection of Ethylene Glycol and Diethylene Glycol in Medicinal Syrups**

**Table of contents**

Fig. S1: PLS quantification models of DEG (A) and EG (B) in glycerol measured through amber PET bottles.

Fig. S2: PLS Latent variables of spiked neat glycerol solution with DEG (A) and EG (B) measurements in amber PET plastic bottles.

Fig. S3: PLS quantification models of DEG (A,C,E) and EG (B,D,F) in spiked representative marketed formulations of (A-B) Benylin, (C-D) Piriteze, (E-F) Calpol measured through amber PET bottles.

Fig. S4: PLS Latent variables of neat propylene glycol measurements in clear glass vials (‘clear’) and amber PET plastic bottles (‘plastic’).

Fig. S5: PLS Latent variables of the finished products measurements in amber PET plastic bottles.

Fig. S6: PLS Latent variables of the finished products measurements through original bottles.

Fig. S1: PLS quantification models of DEG (A) and EG (B) in glycerol measured through amber PET bottles.


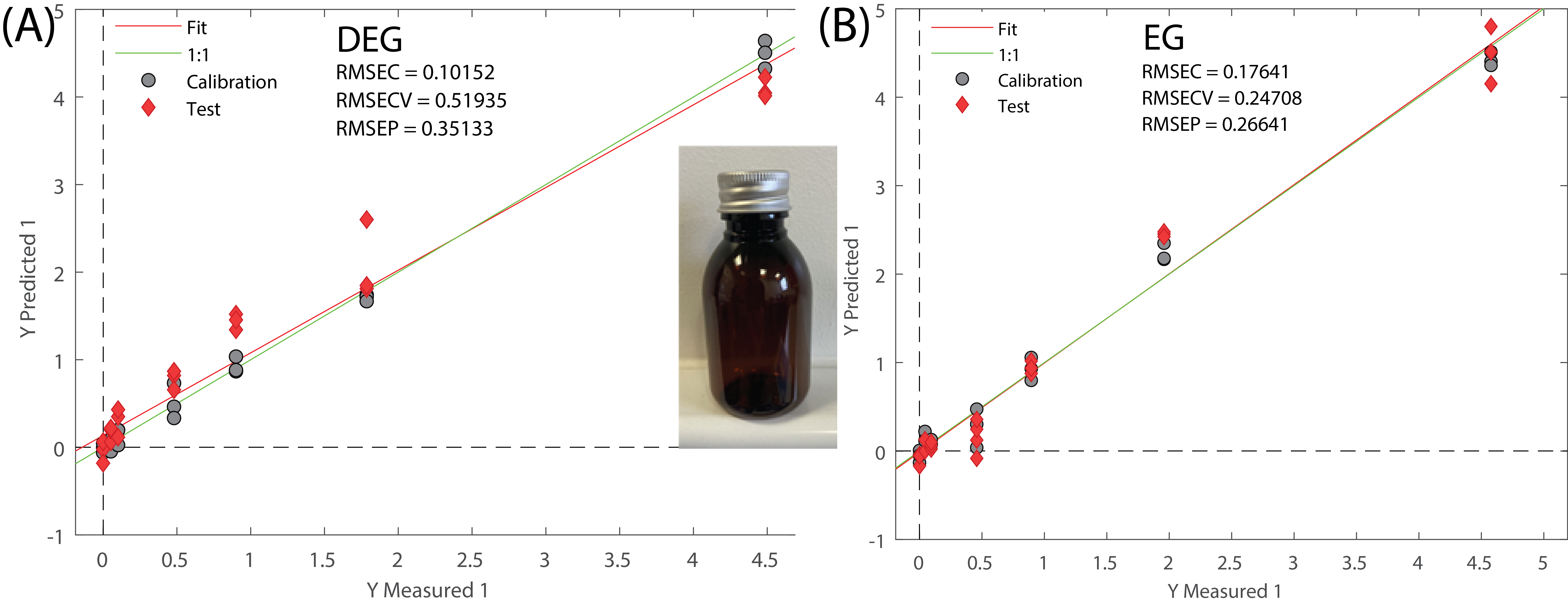


Fig. S2: PLS Latent variables of spiked neat glycerol solution with DEG (A) and EG (B) measurements in amber PET plastic bottles.


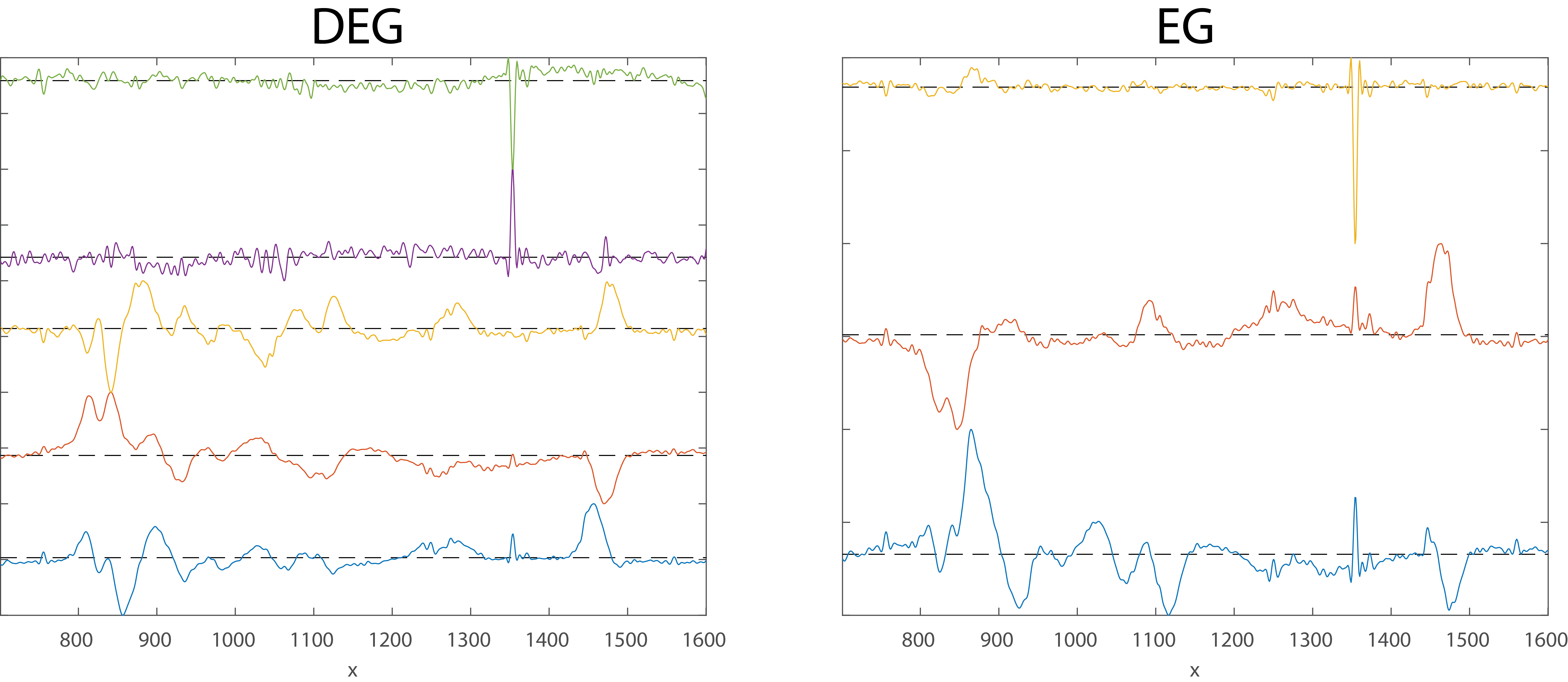


Fig. S3: PLS quantification models of DEG (A,C,E) and EG (B,D,F) in spiked representative marketed formulations of (A-B) Benylin, (C-D) Piriteze, (E-F) Calpol measured through amber PET bottles.


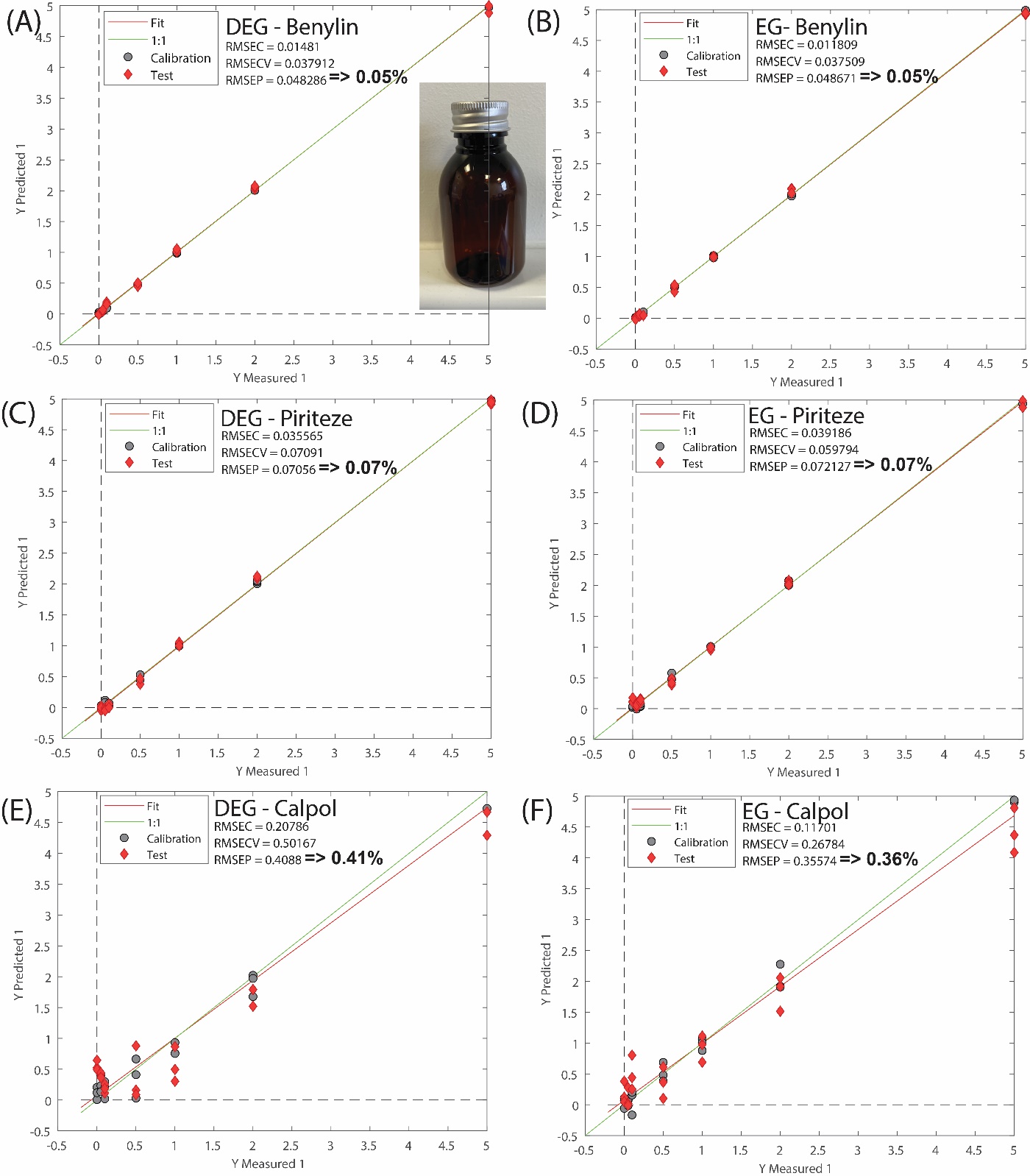


Fig. S4: PLS Latent variables of neat propylene glycol measurements in clear glass vials (‘clear’) and amber PET plastic bottles (‘plastic’).

Fig. S5: PLS Latent variables of the finished products measurements in amber PET plastic bottles.

Fig. S6: PLS Latent variables of the finished products measurements through original bottles.

**References**

[1] WHO, WHO Global Surveillance and Monitoring System for substandard and falsified medical products. Geneva: World Health Organization, (2017). http://apps.who.int/medicinedocs/en/m/abstract/Js23373en/ (accessed April 19, 2025).

[2] T.P. Umar, N. Jain, H. Azis, Endemic rise in cases of acute kidney injury in children in Indonesia and Gambia: what is the likely culprit and why?, Kidney Int. 103 (2023) 444–447. https://doi.org/10.1016/j.kint.2022.12.004.

[3] S. Alkahtani, H. Sammons, I. Choonara, Epidemics of acute renal failure in children (diethylene glycol toxicity), Arch. Dis. Child. 95 (2010) 1062–1064. https://doi.org/10.1136/adc.2010.183392.

[4] J. Schier, A. Chang, V. Kapil, Medication-Associated Diethylene Glycol Mass Poisoning — A Preventable Cause of Illness and Death, N. Engl. J. Med. 388 (2023) 1156–1157. https://doi.org/10.1056/NEJMp2215840.

[5] WHO, Medical Product Alert N°6/2022: Substandard (contaminated) paediatric medicines Substandard (contaminated) paediatric medicines identified in WHO region of Africa, (2022). https://www.who.int/news/item/05-10-2022-medical-product-alert-n-6-2022-substandard-(contaminated)-paediatric-medicines (accessed April 19, 2025).

[6] WHO, Medical Product Alert N°5/2023: Substandard (contaminated) syrup medicines Substandard (contaminated) syrup medicines identified in WHO Region of Africa, (2023). https://www.who.int/news/item/19-07-2023-medical-product-alert-n-5-2023--substandard-(contaminated)-syrup-medicines (accessed January 6, 2025).

[7] WHO, Medical Product Alert N°1/2023: Substandard (contaminated) liquid dosage medicines Substandard (contaminated) liquid dosage medicines identified in WHO European Region and Western Pacific Region, (2023). https://www.who.int/news/item/05-10-2022-medical-product-alert-n-6-2022-substandard-(contaminated)-paediatric-medicines (accessed April 19, 2025).

[8] WHO, Medical Product Alert N°8/2023: Substandard (contaminated) syrup and suspension medicines Substandard (contaminated) syrup and suspension medicines identified in the WHO Regions of the Americas, Eastern Mediterranean, South-East Asia and Western Pacific, (2023). https://www.who.int/news/item/07-12-2023-medical-product-alert-n-8-2023--substandard-(contaminated)-syrup-and-suspension-medicines (accessed April 19, 2025).

[9] M. Ahmed, M. McLeod, J. Nézivar, A. Giuliani, Fourier transform infrared and near-infrared spectroscopic methods for the detection of toxic Diethylene Glycol (DEG) contaminant in glycerin based cough syrup, J. Spectrosc. 24 (2010) 601–608. https://doi.org/https://doi.org/10.3233/SPE-2010-0482.

[10] P. Matousek, I.P. Clark, E.R.C. Draper, M.D. Morris, A.E. Goodship, N. Everall, M. Towrie, W.F. Finney, A.W. Parker, Subsurface Probing in Diffusely Scattering Media Using Spatially Offset Raman Spectroscopy, Appl. Spectrosc. 59 (2005) 393–400. https://doi.org/10.1366/0003702053641450.

[11] S. Mosca, C. Conti, N. Stone, P. Matousek, Spatially offset Raman spectroscopy, Nat. Rev. Methods Prim. 1 (2021) 21. https://doi.org/10.1038/s43586-021-00019-0.

[12] C. Eliasson, N.A. Macleod, P. Matousek, Non-invasive detection of cocaine dissolved in beverages using displaced Raman spectroscopy, Anal. Chim. Acta 607 (2008) 50–53. https://doi.org/10.1016/j.aca.2007.11.023.

[13] S. Mosca, Q. Lin, R. Stokes, T. Bharucha, B. Gangadharan, R. Clarke, L.G. Fernandez, M. Deats, J. Walsby-Tickle, B.Y. Arman, S.R. Chunekar, K.D. Patil, S. Gairola, K. Van Assche, S. Dunachie, H.A. Merchant, R. Kuwana, A. Maes, J. McCullagh, C. Caillet, N. Zitzmann, P.N. Newton, P. Matousek, Innovative method for rapid detection of falsified COVID-19 vaccines through unopened vials using handheld Spatially Offset Raman Spectroscopy (SORS), Vaccine 41 (2023) 6960–6968. https://doi.org/10.1016/j.vaccine.2023.10.012.

[14] Agilent, Resolve Tactical Handheld Raman Analyzer, Mol. Spectrocopy (2018). https://www.agilent.com/en/product/molecular-spectroscopy/raman-spectroscopy/handheld-raman-chemical-detection-systems/resolve-handheld-raman-analyzer-for-through-barrier-chemical-identification (accessed April 19, 2025).

[15] WHO, TESTS FOR DIETHYLENE GLYCOL AND ETHYLENE GLYCOL IN LIQUID PREPARATIONS FOR ORAL USE, Chapter Incl. Int. Pharmacopoeia (2023). https://cdn.who.int/media/docs/default-source/medicines/pharmacopoeia/2023-11-16-deg-eg-inliquidoraldosageforms-qas22-922rev3.pdf (accessed January 6, 2025).

[16] L. MINT, Marion Biotech, maker of toxic cough syrup, loses registration, E-Paper, (2022). https://www.livemint.com/news/india/marion-biotech-maker-of-toxic-cough-syrup-loses-registration-11672421692869.html (accessed January 6, 2025).

[17] REUTERS, S. Widianto, Deadly Indonesian cough syrup was almost pure toxin, court papers show, Reuters (2023). https://www.reuters.com/business/healthcare-pharmaceuticals/deadly-indonesian-cough-syrup-was-almost-pure-toxin-court-papers-show-2023-10-13/ (accessed January 6, 2025).

[18] WHO, Medical Product Alert N°1/2024: Falsified (contaminated) USP/EP PROPYLENE GLYCOL Falsified (contaminated) USP/EP PROPYLENE GLYCOL identified in the WHO Eastern Mediterranean Region, (2025). https://www.who.int/news/item/15-04-2024-medical-product-alert-n-1-2024--falsified-(contaminated)-usp-ep-propylene-glycol (accessed April 19, 2024).

[19] WHO, Medical Product Alert N°4/2024: Falsified USP/EP PROPYLENE GLYCOL Falsified USP/EP PROPYLENE GLYCOL identified in the WHO Eastern Mediterranean Region, (2024). https://www.who.int/news/item/10-10-2024-medical-product-alert-n-4-2024--falsified-usp-ep-propylene-glycol (accessed January 6, 2025).
